# How We Treat Wounds To-day

**Published:** 1891-07

**Authors:** F. S. White

**Affiliations:** Terrell; Assistant Physician State Lunatic Asylum


For Daniel's Texas Medical Journal.

### HOW WE TREAT WOUNDS TO-DAY.

BY F. S. WHITE, M. D., ASSISTANT PHYSICIAN STATE LUNATIC ASYLUM.

[Read before Terrell Medical Society, June 11, 1891.]

WHILE this is not directly in my line of work, yet I have given the subject some thought, have had some little experience and have endeavored to keep abreast with the leaders of modern surgery. Recent investigations into the etiology of pathological processes, have demonstrated the fact that the greater number of the diseases to which animal life is subject, are produced by micro organism. The brilliant results of Koch and his school have established this fact to such a degree of certainty, that it is no longer a matter of controversy with the majority of thinking and learned scientists. The different forms of bacteria, the bacilli, micrococci and spirrilla, are floating in myriads in the atmosphere, disporting in drinking water, and infesting every nook and corner of our habitations, seeking an opportunity when the army of phagocytes, which defend the portals of our life current, is off guard or disabled, to gain an entrance into the very citadel of life, and cripple or put to an end the processes which give the blush of health to the cheek of the maid, and vigor and strength to the sturdy arms of the honest yeomanry of the country. It does not become my subject for me to enter into the consideration of general diseases in any of their aspects. I am limited to the consideration of the treatment of wounds, whether inflicted by the surgeon's knife, or otherwise. Now, the grand principle, the one idea upon which modern surgery rests, and which is kept before every surgeon in red letters, is asepsis and antisepsis. The one thing needful, is to have all wounds asepsic, that is, without any septic or poisonous substance in it; without, in other words, there being present any pathogenic bacteria. This being accomplished, the next thing is to keep it so. This end is obtained by antiseptics. In all wounds in which the nutrition of the parts is not seriously impaired, there will be healing by first intention, if they are kept thoroughly aseptic. Pus cannot form without the pus microbe being present. In order for healing to take place, it is not necessary that there be active inflammation, or in fact any inflammation. It is an exploded theory that healing cannot take place without inflammation.

Union takes place though the medium of the white blood corpuscles, and whenever and wherever there is an injury, they are thrown out in great numbers, and fill up and surround the solution of continuity; and when no extraneous organisms are present to interfere, the process goes on rapidly to restoration. We now come to the all-important question, of when a wound is aseptic, how to keep it so, and when it is septic, how to render it aseptic. The first principle is cleanliness, and is nothing more nor less than keeping out all extraneous material that contains any pathogenic micro-organisms. The directions usually given to accomplish this, are about as follows: Take, for instance, an amputation of a limb. First wash and scrub the parts thoroughly with soap and warm water. Then wash with ether, to remove any oil that may remain; then wash with a solution of bichloride of mercury, of the strength of from 1 to 2500 to 1 to 5000. The operator and assistants should cleanse their hands by the same process. All instruments should be carefully washed and soaked in hot water, and kept during the operation, when not in use, in a solution of carbolic acid. After the amputation is completed, irrigate the stump thoroughly with a solution of bichloride of mercury, 1 to 5000. Stop all oozing; sew up with sterilized silk; put in sterilized draining tube; sprinkle the stump with iodoform; apply over this several thicknesses of iodoform gauze, over this bichloride gauze, over this a layer of cotton and rubber tissue, put on roller bandage, and if the antiseptic precautions have been thorough, healing will take place without a drop of pus, and when the dressing is removed it can remain off. The one symptom, the danger signal which nature throws out when our antiseptic precautions have been unsuccessful, is rise of temperature. When after an operation the temperature rises to 102° or 103°, we know that there is something wrong somewhere, and it is safe to presume that the wound has become septic, and that to such an extent that it is producing systemic disturbance. The treatment under such conditions, is to remove all the dressing, thoroughly cleanse and irrigate the wound again with antiseptic solutions, removing, of course, all effete and unhealthy tissue. Then re-dress with the same precautions as before, and if successful, there will soon be a reduction of temperature. In some instances, however, the cause of the fever may be from some intercurrent source, as malaria, etc. The surgeon is to be the judge, when examining the wound, as to whether or not it is still aseptic. If there is only a drop of pus, and the edges look red and irritated, the trouble is in the wound, and the only proper course is the one above indicated.

Some very eminent surgeons use no antiseptic solutions whatever, only using perfectly pure water. The end sought in either case is the same, and, as I said in the outset, asepsis is nothing but cleanliness. It is but little difference how this is accomplished, but for myself, I prefer to cleanse my wound with antiseptic solution. Very brilliant results can be had by applying this principle to the treatment of abscesses. I have treated quite a number by this plan, with remarkable success. I recall one case which may be of interest: A mammary abscess had been neglected until fluctuation could be detected at two different points. I first washed the breast thoroughly with a solution of bichloride, made free incisions at points where fluctuation was present, evacuated the pus, washed out the cavities with solution of bichloride, applied over each incision a pledget of cotton saturated with same solution. As often as this became dry, it was replaced with a moistened piece. Put on no other dressing, and in a very short time healing, without pus re-forming, resulted.

In treating abscesses of any kind, above everything else do not apply poultices, for they are the pet abomination of modern surgery, and perfect hotbeds for the growth and development of bacteria. If it is considered necessary to soften the skin and underlying tissues, to facilitate pointing, apply some warm antiseptic solution. It is the warmth and moisture that soften the skin, not the mush or bread and milk.

A word more in regard to one or two remedies, and I am done. There are a great many surgeons who oppose the use of iodoform. This I believe is largely due to its odor and association. Most people know that iodoform is a sovereign remedy in venereal sores, and they object to having it used on them for fear some of their friends will think they have been a little indiscreet, and have departed from the strict path of virtue and morality.

It is not claimed for this remedy that it destroys pathogenic bacteria. It only prevents their increase, and this virtually destroys them, for it is by their multiplication and feeding on the tissues that they do harm and set up pathological processes. Sub-iodide of bismuth enjoys quite a reputation with some surgeons, as a dressing, and I can say that on several occasions when I did not desire to have the odor of iodoform, I have used it with good results. Aristol is another antiseptic which is being used extensively, but so far I believe it is limited to the treatment of purulent troubles about the ear and nose. Carbolic acid is an old and tried antiseptic, and to it and bichloride of mercury and iodoform I pin my faith.

